# EHMTI-0054. Monthly variation of Tashkent pediatric headache emergency department visits

**DOI:** 10.1186/1129-2377-15-S1-B9

**Published:** 2014-09-18

**Authors:** S Gazieva, Anna Prokhorova

**Affiliations:** 1 Neurology, Tashkent Medical Academy, Tashkent, Uzbekistan

## 

Headache is the most frequent neurological symptom and the most common manifestation of pain in childhood.

The objective of this article is to determine the monthly variation of emergency department (ED) visits for pediatric headache.

We hypothesized youth have increased headache-related ED visits in the months associated with school attendance.

## Methods

Using a Tashkent representative sample of ED visits in the National Hospital Ambulatory Medical Care Survey from September 2009 to April 2014, we estimated number of visits associated with ICD-9 codes related to headache, migraine, status migrainosus, or tension-type headache in 5- to 18-year-olds. Age-stratified multivariate models are presented for month of visit .

## Results

There was a national estimate 3300 ED visits annually related to headache (2.1% of total visits) in 5- to 18-year-olds. In 5- to 11-year-olds, the adjusted rate of headache-related visits was lower in March (OR 0.42, 95% CI 0.20, 0.88). In 12- to 18-year-olds, there were higher rates in January (OR 1.92, 95% CI 1.16, 3.14) and September (OR 1.64, 95% CI 1.06, 2.55).

## Conclusions

In adolescents we found higher ED utilization in January and September, the same months associated with school return from vacation for a majority of children nationally. No significant reduction in the summer suggests that school itself is not the issue, but rather changes in daily lifestyle and transitions.

No conflict of interest.

